# Surgery

**Published:** 1878-06

**Authors:** 


### Surgery.

Intractable Hernia Reduced by Esmarch's Bandage. *L' Année Médicale* reports two interesting cases of Hernia successfully reduced by Esmarch's bandage. Place, Hôtel Dieu, Caen, France ; Surgeon, M. Dennis Dumont; Assistant Surgeon and Reporter, M. Chapelle.

Case 1.—A. J. B., Journalist, sixty years old. Subject to hernia for ten years. It came down rarely and was returned without difficulty. On the occasion in question it came down the day before he appeared at the hospital, and refused to yield either to the efforts of the patient or the physician.

Examination revealed a left inguinal scrotal hernia, pyriform and about the size of the fist. Color of skin not changed. General state of patient not sufficiently serious to induce the assistant to send for the surgeon—no fever, no vomiting.

A warm bath was administered, extending over an hour, and taxis tried; no result. Injections were employed and compresses of ice water kept over the tumor.

Next day vomiting occurred; general state not alarming. M. Dennis Dumont administered chloroform to complete muscular relaxation. Taxis resorted to but in vain. Esmarch's bandage was then applied as follows: The end was placed over the pubis and held there by the patient; the bandage was then brought down into the left groin under the scrotum, and by three or four circular turns the scrotum was enveloped as far as the penis. Then the penis being enclosed, the bandaging, by means of reverses, was continued pretty tightly as high up as the pubis. The bandage was then secured by means of pins and then carried round the loins.

At the end of an hour the patient had heard the rumbling noise characteristic of reduction. An evacuation of the bowels occurred almost immediately afterwards, and the hernia did not reappear.

Case 2.—A widow, seamstress, forty-two years, constitution pretty robust; entered same hospital Dec. 31, 1877. Had occasionally been troubled with a tumor in the groin for a long time.

It appeared on the least exertion, and disappeared as readily when she assumed the horizontal position. On this occasion it came without the least exertion and could not be forced back as before. She exhibited a left femoral hernia about the size of a hen's egg, hard, and below Poupart's ligament. The abdomen was tympanitic, not painful on pressure, but vomiting had occurred.

The surgeon tried taxis without chloroform, and with chloroform pushed to complete muscular relaxation; no success. The state of patient was not alarming. The elastic bandage was again used thus: A graduated compress was placed over the tumor and a spica bandage applied pretty tight. At the end of two hours the hernia was completely reduced.

This method of the reduction of hernia does not appear to have been adopted much in this country, but in a conversation with Dr. Andrews, he stated that the late Dr. Sherman, of this city, spoke to him of its having been accomplished at least six years ago.

Treatment of Transverse Fracture of the Patella.—At a late meeting of the London Clinical Society, the President, Mr. Callender, exhibited a case of the above treated as follows: A sheet of plaster, fitting the thigh, was made to extend to the upper margin of the patella. Through loops on either side of that bone, extension was made to a canvas slipper, so that the upper fragment was drawn down to the lower. This apparatus was left on after the patient was able to walk about.

[While the above extension was made by pulleys, according to the report, it is probable that some elastic material would answer the purpose equally well, if not better, and at the same time be more simple in design and construction.—Rep.]

Concerning Epithelioma of the Skin.—Busch. (*Arch. F. Klin. Chir.*—B. considers that the first hypertrophic layers of epidermis caused by any irritant, prevent the newly formed epithelial cells from pushing up towards the surface as they should do, and cause their accumulation and development downward. Reasoning thus, he has used a very weak solution of soda to soften the layers whenever he sees signs of incipient epithelioma. The solution is made 1 pt. soda in from 40 to 100 pts. water. After the growth is removed, he uses a still weaker solution as a prophylactic. Busch has succeeded in thus removing growths of this nature, even when an ulcer had formed. He also recommends the same measure for the removal of the epithelial collections often found about the nipples of old women, and for preventing their recurrence.

Intravenous Injection of Milk — A Substitute for Transfusion. — (*N. Y. Med. Jour.*, *May*, 1878.) — Dr. T. G. Thomas, in a paper with the above title, puts forward very cogent reasons why hope should not be abandoned in the class of cases where transfusion of blood, although indicated, is impracticable, until the simple operative procedure of injecting milk into a vein has been tried. He gives first a short *résumé* of the history of transfusion, and then shows that milk is not, after all, so very different from blood, or from the chyle which is poured into the blood. He finds that Dr. Hodder, of Toronto, first resorted to the injection of milk in 1850, in three moribund cases of Asiatic cholera, and that two of them recovered. A year ago Dr. Howe, of New York, pursued the same course in a case of inanition accompanying tubercular disease, but without more than ephemeral success.

Without detailing Dr. T.'s cases, we will only say that he has employed the method in three cases. One made an excellent recovery, the second died of exhaustion, attending suppuration, and the third of prolonged interstitial hæmorrhage. The life of the first was certainly saved, while a longer lease of life was given to the other two than they would have otherwise enjoyed.

Dr. T. alluded to the experiments of Dr. Howe upon dogs, and accounted for his uniform failures on the ground that he had used milk which had stood for several hours; whereas it was most essential that the milk should be absolutely *fresh*. The author sums up with these propositions:

1. The injection of milk is feasible and safe.
2. Only milk removed from a healthy cow, and within a few minutes of its use, should be used. Decomposed milk is as dangerous as decomposed blood.

3. A glass furnished with a rubber tube attached, ending in a very small canula, the whole scrupulously clean, is in every respect preferable to the more elaborate apparatus used in transfusion.

4. The whole proceeding is vastly more simple than transfusion, and offers positively no difficulties.

5. The injection of milk, like that of blood, is commonly followed by a chill, and rapid rise of temperature; these symptoms, however, quickly subside, and improvement follows.

6. The measure is indicated not merely in cases prostrated by hæmorrhage, but in disorders which greatly depreciate the blood, like cholera, pernicious anæmia, typhoid fever, and as a substitute for diseased blood.

7. Not more than eight ounces of milk should be injected at any one operation; this, however may be repeated as occasion requires.

Any accessible vein may be selected; perhaps the cephalic is preferable.

